# A Case of General Dropsy, Successfully Treated

**Published:** 1823-04

**Authors:** Robert Ernest

**Affiliations:** House-Surgeon to the Sheffield General Infirmary.


ART. V.—*A Case of General Dropsy, successfully treated.* By ROBERT ERNEST, M.D. House-Surgeon to the Sheffield General Infirmary.

HAVING read a statement, in the different medical Journals, of the effects of an over-dose of the tincture of digitalis,—a man labouring under asthma having swallowed by mistake, or rather through despair, an ounce of this tincture,—I have been induced to publish the following case, in order to show the surprising effects of small and accumulated doses of this powerful medicine: but, before detailing the treatment, it will be proper to give a history of the patient, his habits, and mode of living, during the last twenty years of his life.

J. C., fifty-two years of age, rather above the middle stature, of a sallow complexion, was born of healthy parents, at Sheffield, in Yorkshire. At the age of nineteen, he was bound apprentice to a scissor-smith, but was only two years at the trade. At the age of twenty-two, he entered into the army; he afterwards remained three years and a half in England, and then sailed, with the 76th regiment, to Bengal. Previous to his arrival at the place of destination, he was seized with the flux,* which disorder continued to trouble him for three months. From August 1797, until October 1817, he enjoyed good health,

* Flux is his own expression; a term, I understand, generally made use of in the army by the soldiers, when they are attacked with either melæna or dysentery.

excepting complaints brought on by irregularities. About this time he was attacked with cholera morbus, of which he was cured within a month. In April, 1819, the regiment embarked for England, and he was much exposed during the voyage to wet and cold weather; and, at the time he arrived at Scilly Islands, he was taken suddenly very ill. The following symptoms will evince the violence of his disorder:—He became generally anasarcous, with a bad cough, his breathing very difficult; he was incapable of lying horizontally; his bowels were costive; his urine scanty, thick, and high-coloured; he was thirsty; his skin hot, particularly during the night; his appetite not much impaired. These symptoms were preceded by languor and rigors.

Occasional purges and mercurials were prescribed for him, which were persevered in until the system became affected, and then they were discontinued; and now the anasarcous swellings had in some degree subsided.

On the 17th of May, he arrived in England, and was sent to the hospital at Fort Pitt, Chatham, where he remained till the 26th of August, when he was removed to London, to pass the Board, and be discharged; upon which, on the 29th of the same month, he was admitted into St. George's Hospital, where he remained until the 8th of October, and was discharged greatly relieved.

He was twenty-five years in Bengal, and his mode of living was very irregular; his diet principally animal food, morning, noon, and night. He frequently drank raw spirits (rum), which however at times he mixed with water; and every now and then he indulged in drinking to great excess, for three or four weeks together. He supposes that he has had syphilitic complaints at least twenty times, and that three attacks were accompanied by buboes and chancres: notwithstanding which, he says that he never was salivated for the cure of this malady.

Immediately after he was discharged from St. George's Hospital, he bent his course to Sheffield, his native place, and was admitted into the Sheffield General Infirmary, on the 29th of October, 1819, in a state pretty nearly approaching to that when he was first seized in April of the same year; but the cough was more urgent, the respiration much more difficult and alarming; the face considerably swollen, and of a livid colour; the pulse not remarkably quick, but accelerated on motion or slight exercise, with a tendency to palpitation of the heart.

*October 29th*, (the day of his admission.) Habeat Bol. c Cal. j.; Pulv. Scillæ, āā gr. iij. hora somni; Pulv. Jalapæ comp. ʒij. mane sumend. Diæta media.—30th. The bolus and powder not having operated, he was ordered ℥ij. mist. purg. statim, et repet. omni hora donec plenè dijecerit alvus.—31st. Has been copiously and freely purged many times, and finds himself much relieved. Pulse the same as on his admission. Hab. Linct. Pectoral urgente tussi. ℞. Sp. Ether. Nitrici, ʒx.; Tinct. Scillæ, ʒiv.; Digital. gr. ij. M. Capt. ʒj. ter indies ex Infus. Juniperi ℥ij.

*November 1st.*—He feels himself better: the medicines to be continued.—2d. He says, he begins to feel full and uneasy as before. Repet. bol. et pulv. purgans; postea, cont. medicament. —3d. The purging medicines having operated freely, he feels relieved; little or no variation in the pulse.—4th. The bowels are open, and he passes more water; complains less of fullness, and is better. Medicine continued.—5th. He says he is much better in all respects; his breathing and cough are much relieved; the bowels are open, and he passes more water. Repet. medicament.

6th.—The pulse, since last evening, has become suddenly and alarmingly slow; he complains of giddiness of the head; has passed from six to eight pints of urine during the last twenty-four hours. He has no thirst, nor are his spirits at all depressed; the body and extremities are of a natural temperature; no nausea, the appetite good, and the bowels are open. Omit the digitalis, &c. Pulse *thirty-two*,* full and strong. ℞. Mist. Camphoræ; Aquæ puræ, ā ℥iijss; Sp. Ammon. comp.; Sp. Lavend. comp. ā ʒiv. M. Capt. ℥jss. ter indies. Cont. Linct. Pectoral. In the evening, the pulse was *thirty*, full and strong.—7th. Not so giddy; in no respect worse; has passed as much urine as the day before. Pulse *thirty;* in the evening 36. —8th. About twelve o'clock this morning, he had occasion to go to the water-closet, and, by the time he reached the doorway, he was suddenly seized with violent giddiness of the head, which greatly affected his sight; and, had he not stood still, he feels assured that he should have fallen down upon the floor. Pulse *forty*. Continue the mixture.—9th. Much better to-day; there is still a great flow of urine; the pulse is *forty-four*, irregular, and intermits about every third stroke.—10th. In all respects

---

* The patient, perceiving that I appeared much surprised at the slowness of his pulse, said, "When I was at St. George's Hospital, the doctors remarked that my pulse was above a hundred; that Mr. Gunning prescribed a medicine that brought it down in five days to twenty-five or thirty beats in a minute. All the medical gentlemen who attended the hospital saw him several times, and counted his pulse; and their opinions of him as to his recovery were very unfavourable. At the end of a week from the time of his pulse becoming so remarkably slow, he began to grow better, and the dropsical swellings began also to subside. During the continuance of the effects of this medicine, his head was very giddy, and he had constant nausea at the stomach, and passed off large quantities of urine."

It is well known that the late Dr. Gregory used to mention, in his Lectures, the case of a young lady, whose pulse suddenly was diminished to twenty-eight pulsations in the minute, from the accumulation of repeated doses of digitalis; and who nevertheless recovered by the free exhibition of brandy.

better; the anasarcous swellings much diminished; pulse the same, and irregular.—11th to the 13th. Going on well: the same medicine continued. On the latter day, pulse in the morning 56, in the evening 68.—No alteration on the 14th.—On the 15th, the patient says he is very well; his breathing is quite free; can lie on either side; he rests well, and coughs very little; the dropsical swellings have now entirely subsided; pulse 64. Continue the mixture.—17th. He now takes exercise, and walks without inconvenience; pulse 76.—18th. The pulse is natural, both as to strength and frequency.—19th. He complains of some dyspeptic symptoms. Omittantur mist. et linctus, et capt. Infus. Anthemid. *c* Potassæ Subcarbon. bis in die.—This was continued until the 26th, when he was discharged, cured.

*Further Observations on this Case.*—I must confess that I was in a state of considerable anxiety and fear for the fate of this patient, when his pulse fell to thirty strokes in the minute; especially as he complained of great weight and uneasiness about the heart and præcordia; symptoms which ought to have been mentioned in the detail on the 8th of November. However, on his informing me that he had been similarly affected before by (as I supposed) the same medicine, my mind was greatly relieved.

I have resided in this Infirmary from its first opening, which is twenty-five years. Having in the course of this time witnessed the sudden and unexpected deaths of several dropsical patients,* after the exhibition of digitalis to its fullest extent, I am very solicitous of calling the attention of my younger readers in particular to the necessity of carefully watching the properties and effects of this powerful and deleterious plant, when exhibited as a remedy in various diseases. In this case the patient took but small doses of tincture of digitalis,—eight or ten drops three times a-day; so that in six days he had taken only three drachms; yet the effects, even from so small a quantity, were truly alarming.

Many other facts might be enumerated, if it were necessary, wherein the most active remedies were almost wholly inert and ineffective: one case of this nature just occurs, with which I will conclude these remarks, fearing that I have already trespassed too much on the pages of a periodical work.

A few years ago, a tall woman, of an athletic make, about forty-five years of age, was admitted into this Infirmary with general anasarca. At this time she passed only small quantities of urine, which was thick and high-coloured; she was also habitually costive. She got her livelihood by going about to

* The patients here alluded to had been labouring for some time under hydrothorax and general anasarca.

public-houses selling pies, was much addicted to drinking spirits, was commonly out late at nights, and much exposed to wet and cold weather. The bowels in a great measure resisted the most active cathartics, both with and without mercury, and it was with the utmost difficulty that free purging could be accomplished. She took calomel and squills in full doses for some length of time; and, at different periods, many of the more drastic cathartics and diuretics, which were persevered in for a considerable time without any apparent effect, or the least amendment in her complaints. It was then judged expedient to omit these medicines altogether, except some cathartic pills which she took occasionally. It will not be a little surprising to learn that these obstinate complaints were cured, in the end, by a domestic remedy. The patient was very desirous of having something that would act as a cordial, and at the same time make her pass more water. It occurred to my recollection that, whenever a friend of mine took two or three glasses of gooseberry wine, (which was made from the green or unripe fruit,) it always acted with him as a very powerful diuretic; I therefore resolved upon giving it a fair trial in this case. I gave the patient a bottle of this wine, and told her to take it at her pleasure. By the time she had finished the bottle, she began to pass copious quantities of water; a second bottle had the effect of removing all the anasarcous swellings, and she continued to pass water freely. In the following week, she was directed to take the compound infusion of gentian, and to repeat the cathartic pills as occasion might require; and in a few days she was discharged cured.

I have been well informed that two other cases of general dropsy have been cured by gooseberry wine, after the exhibition of many active medicines in vain.

*Nov. 28th,* 1822.

---

ART. VI.—*Stricture and Retention of Urine.* By ROBERT ROWLEY, jun. Esq.

