# Errata

**Published:** 1823-05

**Authors:** 


### ERRATA.

Page 298, *for* Pulv. Jalap Comp. ʒij. *read* ℈ij.; *and for* dijecerit *read* dejecerit.

— 298, *for* Digitalis gr. ij. *read* Tinct. Digitalis, ʒij.

